# The hyperemesis gravidarum and pulmonary embolism: A case report and review of literature

**DOI:** 10.5339/qmj.2024.39

**Published:** 2024-07-24

**Authors:** Nissar Shaikh, Umme Nashrah, Roaa Nasser Suleiman, Umm E Amara, Firdos Ummunnisa

**Affiliations:** 1 Surgical Intensive care unit: Hamad medical Corporation/Doha-Qatar; 2 Decan Institute of Medical Science and Research/Hyderabad: India; 3 Department of Anesthesiology, ICU and Perioperative Medicine, Hamad Medical Corporation, Doha, Qatar *Email: rsuleiman@hamad.qa; 4 Apollo Institute of Medical Sciences and Research Hyderabad: India; 5 Haleema Altamimi OBGY Clinic, Doha, Qatar

**Keywords:** Computerized tomography (CT) angiography, deltaparin, dehydration, echocardiogram, hyperemesis gravidarum, pulmonary embolism, pregnancy, starvation, vomiting

## Abstract

**Background:**

Nausea and vomiting occur in more than 70% of pregnant women,^1^ and only 2% of these females progress into hyperemesis gravidarum (HG).^2^ HG is the persistent and excessive vomiting before the 22nd week of gestation. HG patients can develop life-threatening electrolyte disturbances or thromboembolism. Pulmonary embolism (PE) is a thromboembolism that blocks and stops blood flow to an artery in the lung. Both HG and PE increase morbidity and mortality in pregnant patients. HG patients developing PE are reported only in two patients with fatal outcomes in the literature. We report a case of PE in a hospitalized HG patient with a better outcome.

**Case Presentation:**

A 26-year-old previously healthy gravida 3 and para 2 patient was admitted to the Women Wellness and Research Center with HG at 10 weeks of gestation. She developed nausea and vomiting at 6 weeks of gestation and was treated in the emergency department, where she was started on intravenous (IV) fluids for hydration, an antiemetic, and deltaparin for prevention of deep venous thrombosis (DVT), as she was pregnant and dehydrated. She was on potassium replacement therapy for hypokalemia. The patient was improving; still had vomiting, but less frequent. On day 3, following admission, the patient suddenly developed hemoptysis, chest pain, and palpitation. She was tachycardic (120 bpm) and tachypneic (30 breaths per minute). She was feeling dizzy, and her oxygen saturation (Spo_2_) was around 95%. Her chest was clear on examination. Computerized tomographic pulmonary angiography showed bilateral PE. She was admitted to the highdependency unit. The patient was tachypneic and tachycardic and required non-invasive ventilation. A therapeutic dose of enoxaparin (1 mg/kg) was started and supplemented with fentanyl plus paracetamol for analgesia, continued IV fluids, and promethazine.

Her respiratory symptoms and tachycardia improved by day 6, she was transferred to the ward from there and discharged home by day 10, on enoxaparin therapeutic dose (1 mg/kg), and follow up in outpatient clinics showed no issues, and she is doing fine.

**Discussion:**

HG is a severe clinical disease in pregnancy where patients have intractable nausea and vomiting with increased morbidity and even mortality. These patients frequently present with ketonuria, dehydration, electrolyte abnormalities, and a weight loss of 7%. Rarely, these patients’ present with severe vitamin deficiency, causing a neurological emergency called Wernicke’s encephalopathy. The occurrence of DVT is one of the main risk factors due to prothrombotic conditions in pregnancy in combination with dehydration in these patients. The occurrence of PE is reported in two cases of HG in the post-mortem. Our patient developed bilateral PE, a medical emergency due to immobility, dehydration, and prothrombotic predominance during pregnancy. PE was detected early and managed, leading to a better outcome.

**Conclusion:**

HG should be diagnosed early, followed by admission of the patient to the hospital. Our patient with HG was complicated by a rare bilateral PE due to a combination of pregnancy, dehydration, and immobility, despite DVT prophylaxis with a favorable outcome. Clinicians should have an index of suspicion for DVT and PE in these dehydrated pregnant patients. A high index of suspicion, early diagnosis, and management by a multidisciplinary team are key for better outcomes of PE in our HG patient.

## Background

Pregnancy is an important period that causes physiological, anatomical, and social changes. Any adverse event or complications during this period carries a huge psychological impact. Hyperemesis gravidarum (HG) is one of these clinical events.^1^ HG is defined as “persistent and excessive vomiting before the 22nd week of gestation.”^2^ HG is also termed “pernicious” vomiting of pregnancy, which is a severe form of nausea and vomiting during pregnancy, particularly in the first trimester, requiring hospitalization and treatment.^3^ The majority of pregnant females have nausea and vomiting during pregnancy, and only 0.3% to 2% will have HG^1,2^. The most frequent causes of hospitalization in HG patients are due to dehydration, ketonuria, electrolyte imbalance, and arrhythmias.^4^ HG can be life-threatening if it gets complicated into Wernicke’s encephalopathy, stroke, acute kidney and hepatic injuries, vitamin K deficiency and coagulopathy, esophageal injury, pneumomediastinum and pneumothorax, deep venous thrombosis, and pulmonary embolism (PE).^4^ If complications from HG are not identified and treated early, the disease may indeed be fatal. In up to 2% of pregnant patients in African nations, HG can result in death.^3^ In this era of exploring the role of micronutrients and antioxidants in neurological diseases, vitamin deficiencies in HG, particularly Wernicke encephalopathy, occurs, which is a neurological emergency.^5^ Similarly, HG can cause PE, which is a medical emergency. The occurrence of PE in HG is rarely reported in the literature. Keskinkilic et al. reported fatal PE in two patients with HG.^6^ We report a case of PE in an HG patient with a favorable outcome, and multidisciplinary team management with timely diagnosis and management.

The publication of this case report was approved by Hamad Medical Corporation’s Medical Research Center (MRC-04-23-626), and written consent was obtained from the patient to publish the case.

## Case Presentation

A 26-year-old gravida 3, para 2, with a history of lower section cesarean section due to prolonged 2nd stage of labor in her second pregnancy. The patient had repeated visits to the emergency department for nausea and vomiting; she was treated with oral antiemetics and discharged home. In her 10th week of gestation, she was taken to the hospital by ambulance as she had intractable vomiting; her laboratory investigations showed hypokalemia (serum potassium = 3.1 mmol/L), ketonuria (+4), and she was dehydrated. The patient was admitted to the ward and started on intravenous (IV) fluids (Ringer lactate 100 mL/hour), promethazine 25 mg every 6 h and deep venous thromboprophylaxis with deltaparin 5,000 IU subcutaneously once daily. Otherwise, she remained hemodynamically stable.

**Figure 1.**
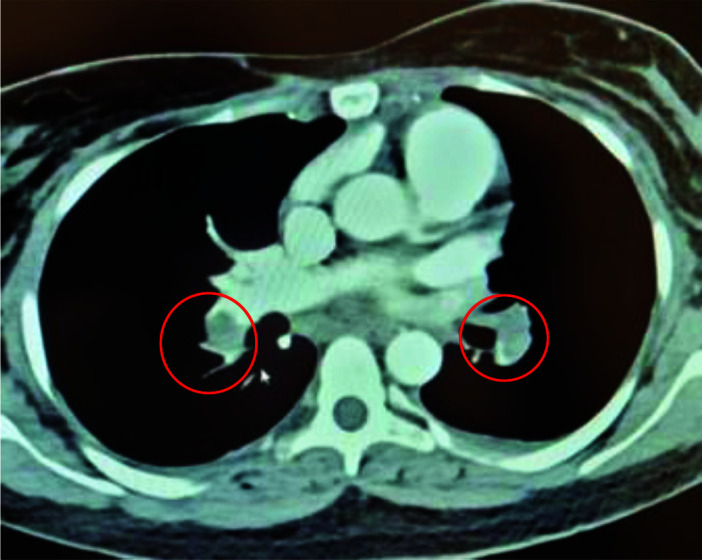
Computerized tomographic pulmonary angiogram (CTPA) showing bilateral pulmonary embolism marked by circles.

On day 3 of admission, she complained of chest pain with breathing difficulty, palpitation, and hemoptysis. She was tachycardic (110 to 120 beats/min), tachypneic (28 to 30/min) feeling dizzy, and still vomiting. Details of the patient’s vital signs and laboratory parameters are described in Table 1. Immediate computerized tomographic pulmonary angiography (CTPA) showed bilateral pulmonary embolism (Figure 1). In Figure 1, bilateral pulmonary embolism is marked by circles. She was transferred to the High Dependency Unit, started on a therapeutic dose of enoxaparin (1 mg/kg) and required non-invasive ventilation. Her echocardiogram showed PE with mild straining on the right ventricle. Along with supportive care, we added fentanyl and paracetamol for analgesia to the therapy. Both of her lower limb’s ultrasound doppler scans were normal. With continued resuscitation and above-average therapy for the next 4 days, she progressively improved. Her tachycardia and tachypnea settled, and oxygen saturation was maintained on room air (96%–97%). She was transferred to the ward on day 6, then discharged home by day 10, on a therapeutic dose of enoxaparin (1mg/kg). The patient is followed in outpatient clinic at 3, 6 months and is doing well without any complications or complaints.

## Discussion

HG is associated with morbidity and mortality. In the 1940s in the United Kingdom, it caused 159 deaths per million pregnancies, which continued to decrease, and by the early nineties, three HG patients had died, two due to Wernicke encephalopathy and one due to aspiration.^7^ Hence, Wernicke’s encephalopathy remained a neurological emergency in HG patients.^5^

There are various risk factors described for the occurrence of HG, including primigravida, nonsmokers, respiratory and urinary tract infections, high intake of saturated fats, multiple pregnancies, and female fetuses.^8^

Various etiologies are described for the occurrence of HG, namely human chorionic gonadotropins, estrogens, *Helicobacter pylori* infection, and psychological factors.^9^

There are no specific criteria for the diagnosis of HG; it is commonly diagnosed by persistent vomiting (more than three times in a day) not associated with any maternal diseases, dehydration, or electrolyte abnormalities. HG patients frequently present with ketonuria. HG patients may present with Wernicke’s encephalopathy with a triad of ophthalmoplegia, confusion, and ataxia.^5^ HG can be classified into mild, moderate, and severe depending upon nausea and vomiting, and utilizing a pregnancy unique quantification of emesis and nausea score index.^10^

The management of HG includes both nonpharmacological and pharmacological therapies. Consumption of bland food, frequent small meals, and avoiding spicy food and ginger is often considered a helpful remedy for alleviating nausea and vomiting. Ginger contains compounds such as gingerol and shogaol, which have anti-inflammatory and antiemetic properties that may help to reduce nausea and vomiting.^11,12^ Pharmacological treatment is mainly anti-emetics, which are safe in pregnancy. If the HG patient has persistent vomiting for weeks, causing weight loss, ketonuria, electrolyte disturbance, and dehydration, then early hospitalization is highly recommended.^11^ IV fluids containing 0.9% saline or Ringer’s lactate solution are commonly used to rehydrate patients with HG. These fluids help restore electrolyte balance and maintain hydration levels. Dextrose-containing fluids are generally avoided initially because they can exacerbate vomiting and Wernicke encephalopathy.^11^ Thiamine should be administered intravenously for the first 3 days of hospitalization to ensure adequate levels are maintained and to prevent Wernicke encephalopathy. The Royal College of Obstetricians and Gynecologists green top guidelines included HG as a risk factor for thromboembolism.^12^ The risk of thromboembolism in HG patients is mentioned in the majority of HG literature without documenting the actual incidence or prevalence.^11,12^

PE can be life-threatening and is a medical emergency rarely reported in HG patients. PE is one of the leading causes of maternal death in developed countries.^13^ The risk of PE is increased throughout pregnancy due to anatomical, physiological, and hormonal changes during pregnancy and the post-partum period.^14^ The risk of venous thromboembolism increased by 4 to 5-fold during pregnancy and is highest in the post-partum period (increased by 20 to 80-fold).^6,15,16^ The occurrence of PE in HG with favorable outcomes is not reported. However, two fatal cases of PE in HG patients were reported in the literature, and in both cases the diagnosis was not suspected and delayed.^6^ Life-threatening complications are rare in HG patients, and the most common one is Wernicke’s encephalopathy. Pope et al., in their review of more than a thousand HG patients with life-threatening complications, did not report a single case of PE.^4^

Our patient was hospitalized, dehydrated, and had ketonuria with limited mobility. These factors, in combination with the prothrombotic risk of pregnancy, may have complicated into a PE.

During pregnancy, PE is underdiagnosed, and diagnosis is often delayed due to the fear of radiation exposure and its adverse effects on the fetus and mother. Imaging studies, particularly CTPA, is highly sensitive and specific in diagnosing PE; it is also useful in excluding PE and determining other diagnoses. CTPA gives alternative diagnoses.^16^ Hence, it is suggested to use CTPA to diagnose PE early and start therapy earlier. If needed, one can cover the abdomen during CTPA with a lead sheet to avoid the radiation effects on the fetus.

When PE occurs in the immediate peripartum or intrapartum period, it is recommended to use unfractionated heparin, and in the ante- and post-partum periods, low-molecular-weight heparin (LMWH) is the drug of choice due to its advantages of safety, efficacy, and ease of administration.^14,16^ The use of warfarin is contraindicated in pregnancy due to its teratogenicity.^17^

## Conclusion

PE is rarely seen in HG patients. HG patients are at risk of developing PE, so an index of suspicion should prevail with the required diagnostic tests. Our patient had dehydration, immobility, and prothrombotic state of pregnancy that predisposed her to the development of PE. Even though the patient was on a prophylactic dose of LMWH. A high index of suspicion, early diagnosis, and management by a multidisciplinary team are key for better outcomes of PE in our HG patient.

## Conflict of Interest Statement

All authors declare that they did not have any financial or academic or any other conflict of interests.

## Figures and Tables

**Table 1.** Showing patient’s vital signs and laboratory parameters.

**Parameters**	**Day 2**	**Day 3**	**Day 4**	**Day 5**
Heart Rate (beats per minute)	88	120	105	90
Respiratory Rate (breaths per minute)	20	30	26	22
Oxygen saturation (Spo_2_ %) on 3 L oxygen	97 (room air)	95	96	97
Serum potassium (mEq/L)	3.1	3.6	4.0	3.8
White Blood Cell × 10^3^/microlitres	11.3	15.9	12.6	10.2
Haemoglobin (g/dL)	13.9	13.9	13.3	13.0
Prothrombin Time (seconds)	13.7	12.1	11.7	12.0
INR	1.0	1.0	1.0	1.0
Activated partial prothromboplatin time (seconds)	34	30	32	30
Platelet count × 10^3^/microlitre	316	325	360	319
